# Forced Respiration—Case No. 3

**Published:** 1888-03

**Authors:** Geo. E. Fell

**Affiliations:** Professor of Physiology and Microscopy, Medical Department, Niagara University, etc., Buffalo, N. Y.; No. 72 Niagara Street


### *FORCED RESPIRATION—CASE No. 3.*

By GEO. E. FELL, M. D., F. R. M. S.,

Professor of Physiology and Microscopy, Medical Department, Niagara University, etc.,
Buffalo, N. Y.

At the date * of this writing, four lives have been saved by this operation, viz., three in Buffalo by myself, and one in Vienna.

The first case † was that of Mr. P. B., Buffalo. He had taken twenty grains of morphine and some chloral hydrate

* February 15, 1888.
† This case was fully reported in the November (1887) number of this journal.

previously; this was entirely absorbed. The known methods of resuscitation had been tried, and failed. After the pupils had dilated in the last stage of asphyxia, *forced respiration* was resorted to, and the patient is now alive and well.

The second case was cited in the *Lancet* of October 15, 1887. Dr. Länger, of the Vienna Hospital, took an over-dose of morphine or laudanum. An incision was made in the trachea, and the patient's life was saved by *forced respiration*. I have not seen the details of this case. The operation was made after the reports of my first case (read before a section of the Medical Congress at Washington,) had appeared in the medical journals.

The third case, my own, is the subject of this paper.

The fourth case, also brought to a successful termination through my labors, is yet to be reported.

REPORT OF THIRD CASE.

Mr. J. A. V., aged forty-three, a man of family, pleasantly situated, but subject to fits of melancholia and troubled with insomnia, took an over-dose of laudanum* and chloral about **9.00 or 10.00 P. M.**, Saturday, **December 10, 1887.** He had previously taken a bath, changed his underwear, and then retired. About midnight, his wife heard him breathing heavily, tried unsuccessfully to arouse him, and sent for a physician.

Dr. Lawrence G. Hanley, of the Emergency Hospital, was the first to respond to the call, and was shortly thereafter followed by Dr. Jacob. The condition of the patient at this time, **1.15 A. M.**, indicated that a large dose of some powerful narcotic had been taken. Breathing was stertorous; pulse, 128; respirations, six per minute, and pupils contracted. At 1.40 A. M., Saturday morning, I was called. Quickly arriving upon the scene, I found the patient upon the floor of his bed-room; the physicians were employing Sylvester's method of *artificial respiration*. Assuming, at their request, entire charge of the case, I had the patient placed upon a mattress on the dining-room table.

* Two ounces of laudanum were taken, and all retained.

**2.20 A. M.**—The natural respirations ceased, or would last but a short time without the aid of the *artificial respirations.* Pulse, seventy-two to eighty-four, indicating satisfactory oxygenation of the blood; however, the notes * taken at the time show that the natural respiratory efforts were so irregular and deficient that it was difficult to count them.

The inefficient character of the natural, even when supplemented with the *artificial respirations of Sylvester*, were evidenced by the gradually marked increase of cyanosis. Previous to this, when noticing the first good results of the *artificial respiration* in this case, I informed the physicians that this would be a good time to effectually answer all skeptical "wise-acres" who believed that *artificial respiration* would save life under conditions of *deep* narcosis from poisons which act upon the respiratory centers. I informed those present that if the life of the patient could be saved by *artificial respiration*,† or any other *known* means, my apparatus *should* not be used.

It was evident that the *artificial respirations* were doing little good, growing less and less efficient.

**2.30 A. M.**—Natural respirations, seven per minute.

**2.40 A. M.**—Natural respirations, stertorous, twelve per minute; pulse, 120.

**2.45 A. M.**—Natural respirations, fifteen per minute, but so superficial or "shallow," that little good was effected by them.

### FAILURE OF ARTIFICIAL RESPIRATION.

**3.25 A. M.**—Respirations failed. Owing to evident signs of heart failure, and the uncertainty of its ability to stand the strain to which it was being subjected, it was considered by all the physicians present that the life of the patient demanded the application of *forced respiration.* Time was given to demonstrate beyond question the uselessness of the *artificial respiration*,

* This case is reported from full notes taken during its progress by the different physicians present.

† By *artificial respiration* is understood the various methods ordinarily used, as Sylvester's, Marshall Hall, and others. *Forced respiration* is the name I have given my operation.

until it was feared the patient might succumb before the *forced respirations* could be applied.

**3.40 A. M.**—Operation of tracheotomy begun. Blood venous; Dr. Hanley remarked, at the time, that it was "ebony-colored."

### FORCED RESPIRATION SUBSTITUTED.

**4.05 A. M.**—*Forced respirations* begun. In a short time, the pulse became stronger, and was reduced to seventy-eight per minute.

**5.30 A. M.**—Pulse, 102.

**5.45 A. M.**—Pulse, sixty-four.

**6.25 A. M.**—The patient, *up to this time insensible*, opened his eyes, stared in a half-dazed manner, and raised his head from the pillow. He recognized Dr. Goldberg, (by voice only, as afterwards stated,) and, in answer to enquiries, stated that he had taken twenty grains of chloral with some stimulant. This was found to be untrue.

### INTERESTING PHYSIOLOGICAL PHENOMENA.

**6.45 A. M.**—First noted, that when *forced respiration* is discontinued, not the slightest attempt at breathing is made by the patient. This was not produced by a condition of *apnea*, but under the ordinary continuous operation of the apparatus; furthermore, owing to the complete paralyzation of the respiratory centers, this condition prevailed when the face, lips, eyelids, ears and back of neck were in a state of cyanosis; when it would seem that all the tissues of the body would be crying for oxygen, and yet, when the *forced respirations* were *discontinued*, not the least effort would be made toward a respiratory act on the part of the patient. But the most remarkable act in this drama of life was, that under this condition of marked paralysis of the respiratory centers, the patient was *conscious*, and would respond to ordinary requests, such as drinking when desired to, and also when asked to "*take a long breath,*" would evidence the power of the will over the respiratory muscles or centers by responding with a good effort at inspiration. He would, however, immediately subside into his former lethargic state, and make not more than the one voluntary effort at respiration. When the condition of asphyxia had been reached, the respiratory efforts were noted, but immediately subsided when the *forced respiration* was again instituted. I will not make any comments on these phenomena at this time, as I propose to consider them in connection with these three cases in another paper. Many persons, besides the four physicians who were present, observed them.

During the progress of the case, water was frequently swallowed by the patient. In one or two instances, the *forced respirations* were unintentionally kept up when the patient was swallowing; the glottis being opened at this time, water entered the lungs and was subsequently coughed up and passed out of the valve of the apparatus.*

**7.00 A. M.**—Pulse, ninety-six.

**8.15 A. M.**—Pulse, 108. It was found that the patient could breathe for himself, but only for a short time, and the *forced respirations* had to be continually kept up.

**9.00 A. M.**—The trachea tube not being secured tightly in the trachea, permitted quite an amount of blood to pass into the lungs. This gurgled ominously at each inspiration. The loose tube also allowed the air to pass upwards into the mouth, so that the lungs were not thoroughly inflated in the inspirations. With a curved needle encircling the trachea, another ligature was passed and tightened about trachea and tube. The *forced inspirations* following, markedly improved the action of the heart.

As the poison become more completely incorporated with the blood, through absorption in stomach and intestines, the effect of even a short stoppage of the *forced respirations* was indicated in

* This indicates, in part, the value of the application of the apparatus in cases of drowning, and also that it would be objectionable to pass a tube into the larynx by way of the buccal cavity when the elimination of poison is important.

a weaker action of the heart. At one time, the rubber tube connecting the respiratory valve with the trachea tube, became almost completely clogged with clotted blood. It was removed and thoroughly cleaned, as was also the inner tube of the tracheotomy tube a number of times. Digitalis fluid extract, in M. ss. doses, was given a number of times; also atropia, one-eighth grains at one time, and smaller doses also. No dilatation of the pupil took place at this time.

The question of keeping up the *forced respiration* when there seemed to be no prospect of the ultimate recovery of the patient, was *seriously* discussed. At one time, I stopped them for a longer period than usual, thoroughly discouraged and tired. The man was not dead, and we *had to keep it up.*

GIVEN UP TO DIE.

**11.30 A. M.**—Drank some brandy and water; vomited. As the patient had at this time been given up to die, his family were permitted to see him and "bid him good-bye."

**12.00 M.**—Pulse, 117. Grains 1-75 atropia administered hypodermically.

**12.10 P. M.**—Face cyanosed; efforts to breathe made; twitching of toes; respirations not supplying enough air.

**12.40 P. M.**—Owing to a solution of atropia being placed on or in the eyes, the pupils gradually dilated.* Pulse, 126.

**12.55 P. M.**—The patient, who had become unconscious for a short time, regained consciousness, and drank some water. Pulse, after drinking, 168, weak and flickering. After this, more air was administered, and three movements of the bellows used for the inspirations instead of two, as formerly.

† The Rev. Mr. Gracey, of Grace M. E. Church, looked in upon the operation about this time; remarked he could do

* This may not have been judicious, but was done under the positive belief of all physicians present that the patient could not survive.

† Even in Buffalo, where I have been known, I trust, as a truthful man, some physicians have questioned some of the facts stated regarding the first case of *forced respiration.* I introduce these circumstances here to indicate the serious aspect of the case as held by all acquainted in any way with it. The reports of my former case and this one are, so far as the serious condition of the patient is concerned, conservative.

no good there, and held a service of prayer in the adjoining room.

**3.20 P. M.**—Temperature, 100.5 Fah.

**6.00 P. M.**—Pulse, 120.

PATIENT BEGINS TO BREATHE FOR HIMSELF.

**6.15 P. M.**—Respiration, fourteen per minute. Patient began breathing for himself. This lasted fifty-five minutes, when the respirations lowering to eight per minute, *at the request of the patient*, the *forced respirations* were again proceeded with.

**9.15 P. M.**—Pulse, 120; respirations, fourteen, natural; becoming shallow, they were supplemented with the *forced respirations.*

**11.30 P. M.**—Pulse, 100.

**December 11, 1887, 12 Midnight.**—It is now twenty hours since the forced respirations were begun.

**1.05 A. M.**—Pulse, 128, strong. The patient has been breathing for himself for the last four hours, but has *now requested* that the *forced respirations* be used for a time. Since then, he has breathed for himself. For over fourteen hours, he could not be left to breathe for himself, even for a half-minute, without evident discomfort and danger, viz., between 4.00 A. M., 10th inst., and 6.30 P. M., 10th inst., and for nearly seven hours thereafter the *natural* had to be supplemented by the *forced respirations.*

**4.00 A. M.**—Pulse, 117. Although *oleum tiglii to gtt. v.* have been administered, no movement of the bowels have taken place.

Essence of pepsine, beef peptonoids, milk and spirits frumenti given a number of times. Enemata of water, soap and water with oil and stimulants, given also.

CATHETERIZATION.

Every six or eight hours catheter used. Up to 12.30 A. M., 11th inst., nearly twenty-four hours after the patient was first discovered, and some twenty-seven hours after two ounces of laudanum had been taken, not more than six ounces of urine had been drawn from the patient. This large amount of poison (two ounces) had been undergoing the round of the circulation, producing its maximum effect on the whole system. The left arm was partially paralyzed, the brain congested.

Between 3.00 and 4.00 A. M., 11th inst., bowels moved for the first time. At 7.00 A. M., the patient left the table, without assistance, to use stool. At 9.00 A. M., the tracheotomy tube was removed, wound plugged antiseptically, and patient put to bed.

Although very seriously ill for three or four days following, no serious lung difficulty set in, the patient has fully recovered, and regrets the condition of mind which influenced or controlled him at the time of the deed. Furthermore, he has made up his mind to manfully battle through the rest of life, and deserve the kind consideration of his fellow-men.

### APPARATUS USED.

The apparatus used in this case is the modified form described in a former paper (see below); and owing to its admirable practicability, as evidenced in this most extended case, I shall recommend it until further improvements may suggest themselves.

The simplicity of the apparatus will permit it to be used by any one of ordinary intelligence upon proper instruction, and after the tube is inserted into the trachea. A simple bellows, arranged to produce a steady stream of air, and worked by hand or foot-power, is connected with the valve of the apparatus by a rubber tube. The valve connects also with the tube in the neck by another rubber tube, which is easily attached at the trachea end. The surgeon who controls the valve is guided for the time of inspiration, say by two or three movements of the bellows. By this means, over-inflation of the lungs is prevented. Although the apparatus has been used steadily from two and one-half hours in the first case, to twenty and one-half in the last, no serious condition of the lungs has been produced. Marshall Hall's objections to "the use of bellows or any forcing apparatus" falls flat before such testimony.

Electric-motor power, to keep the air-forcing apparatus working, might be substituted for hand-power.

My thanks are due to all physicians, students and laymen who for hours assisted in keeping the life in the body of our patient. To know that our work is gratefully appreciated by the one whose life was saved, is also cause for congratulation to all engaged in the case.

For further information regarding *forced* respiration, see my paper in this journal of November, 1887.

No. 72 Niagara Street.

[On page 346, third line in last paragraph should read "by Dr. Jacob Goldberg." Dr. Samuel Goldberg was present later in the case.]

